# Pre-participation gynecological evaluation of female athletes: a new proposal

**DOI:** 10.1590/S1679-45082014AO3205

**Published:** 2014

**Authors:** Tathiana Rebizzi Parmigiano, Eliana Viana Monteiro Zucchi, Maíta Poli de Araujo, Camila Santa Cruz Guindalini, Rodrigo de Aquino Castro, Zsuzsanna Ilona Katalin de Jármy Di Bella, Manoel João Batista Castello Girão, Moisés Cohen, Marair Gracio Ferreira Sartori

**Affiliations:** 1 Universidade Federal de São Paulo, São Paulo, SP, Brazil.

**Keywords:** Female athlete triad syndrome, Urinary incontinence, stress, Premenstrual syndrome, Athletic performance, Questionnaires

## Abstract

**Objective:**

To propose the inclusion of a gynecological investigation during the evaluation of athletes before competitions, using a specific instrument called the Pre-participation Gynecological Examination (PPGE).

**Methods:**

The study assessed 148 athletes, mean age of 15.4±2.0 years, who engaged in eight different sports modalities, and who responded to a questionnaire named Pre-Participation Gynecological Examination (PPGE), to the International Consultation on Incontinence Questionnaire - Short Form (for urinary loss), and to the Eating Attitudes Test (for eating disorders).

**Results:**

Fifty percent of the participants reported irregular menstrual intervals, 23.0% did not know about sexually transmitted diseases, and 72.4% denied having, at least, an annual gynecological appointment. The study identified 18.2% who had urinary loss, and 15% presented with an increased risk of eating disorders. Moreover, 89.9% were not familiar with the occurrence of urinary incontinence in athletes and did not know that they were susceptible to the female athlete triad. A total of 87.1% of them stated that would not mention these issues to their coaches even if this would improve their health or performance.

**Conclusion:**

The Pre-Participation Gynecological Examination can be considered an easy-to-apply instrument that allowed the diagnosis of alterations often underestimated by the athletes themselves. After its application, the alterations were identified, and determined the athletes’ referral to appropriate evaluation and treatment.

## INTRODUCTION

Sedentary life is related to an immense quantity of health risks. Conversely, people exposed to an extremely active lifestyle or athletes at a competitive level are also susceptible to a significant number of threats to their health and well-being, especially in the absence of adequate medical supervision.^(1)^

It is known that a few alterations can be preexistent and others, exacerbated during engaging in sports. For this reason, we propose the performance of a pre-participation evaluation – called Pre-Participation Examination (PPE) – for all the individuals willing to initiate or maintain a physical exercise routine. The objective of the PPE is to promote health and safety of all sportspeople in training and competitions, and not exclude them from the activities.^(2)^

In this context, despite the fact that since 2007 the American College of Sports Medicine (ACSM) recommends the importance of the PPE specifically in female athletes, this practice is not common, especially in our country. PPE is not implemented in an adequate or uniform manner, and therefore efforts are great for the establishment of protocols.^(3)^ It was only in 2010 that for the first time specific care for female athletes was officially proposed by means of investigation of issues that may affect their health and their participation in sport activities.^(4)^

The assessment of some characteristics of the menstrual cycle such as age at menarche, date of last gynecological visit, presence of amenorrhea for more than 6 months, and acknowledgement of the association between menstruation and physical performance, has long been the object of study. It should be mentioned that, in some cases, the PPE may be the only contact the athlete has with a physician, increasing even further the importance of this evaluation.^(5)^

With increasing female participation in sports, it is indispensable that some issues, such as the female athlete triad, premenstrual tension (PMS), and urinary incontinence, be informed to sportswomen, whether they are of the elite or not. Many women, just as the professionals that oversee their well-being and training, are unaware of the changes to which they are susceptible, and they do not recognize that they are placing at risk their health, and on a second plane, their sports performance.^(6)^

Rumball and Lebrum et al.^(6)^ suggested the need to reserve a specific portion of the evaluation for issues inherent to the female body, with specific and focused gynecological and nutritional investigations, thus avoiding that these themes be omitted by these women or that their care be underestimated by the professionals involved. This is in agreement with a study published by Careck and Futrell, which points out that women do not feel comfortable in answering questions that involve gynecological aspects, use of alcohol and drugs, and eating habits in the traditional form of evaluation.^(7)^

In Brazil, as in the rest of the world, the *Sociedade Brasileira de Medicina do Esporte* [Brazilian Sports Medicine Society] also recognizes the metabolic differences between men and women and takes position regarding the particularities of women who engage in physical activity without, however, presenting any specific protocol until now for this care.^(8)^

Gynecological questions are not a part of the conventional pre-participation evaluation, characterized exclusively by the coverage of orthopedic and cardiological aspects. The tool presented is the first within a national context that values this specific investigation and that proposes its inclusion at this point of the evaluation.

## OBJECTIVE

To propose the inclusion of the gynecological investigation during pre-participation assessment of women who engage in physical exercise, by means of a specific tool called Pre-Participation Gynecological Examination (PPGE).

## METHODS

The study was carried out at the *Centro Olímpico de Treinamento e Pesquisa *– COTP [Olympic Training and Research Center], located in São Paulo (SP), during the first semester of 2010. This is a venue recognized as a training center for athletes of ten different sports modalities, three of them exclusively female (soccer, handball, and basketball), six mixed (wrestling, judo, track and field, gymnastics, swimming, and boxing), and one, male (volleyball). Its medical team was the first in the country to include a gynecologist specialized in female athlete care.

The project was approved by the Research Ethics Committee of the *Escola Paulista de Medicina da* *Universidade Federal de São Paulo*, with the title *Avaliação ginecológica da mulher atleta*, registration number 1.772/10 with the committee, and by the *Coordenadoria de Gestão do Esporte de Alto Rendimento* [Management Coordination of High-Performance Sports], of the COTP.

It was proposed that, pending on the filling out of an Informed Consent Form by the person in charge, information lectures be given related to female health and aspects associated with the female sportsperson, such as female anatomy, physiology of the menstrual cycle, contraceptive measures, sexually transmitted diseases (STDs), uterine cervix cancer prevention test, female athlete triad, urinary incontinence in athletes, premenstrual tension (PMS), and the relation between contraceptives and doping. Inclusion criteria for participation in the lectures: age over 13 years, duly signed parental consent, and at a time compatible with annual renewal or first time of the pre-participation evaluation.

A total of 148 athletes participated in the lectures, with a mean age of 15.4±2.0 years, and they practice eight different sports modalities that women at COTP engage in.

After the lectures, questionnaires were distributed to all athletes to collect individual information on gynecological history. This questionnaire was called the “Pre-Participation Gynecological Examination”. The model proposed was prepared by the first author of this study; it is self-reported and does not depend on supervision for its completion (Appendix 1).

At the end, the International Consultation on Incontinence Questionnaire-Short Form (ICIQ-SF) for evaluation of involuntary urine loss, validated and translated into Portuguese, was attached,^(9) ^as well as the Eating Attitudes Test^®^ (EAT-26), for eating changes.^ (10)^ Posteriorly, all information obtained was attached to the medical records of the Medical Department of the *Centro de Excelência em Medicina Esportiva Caio Pompeu de Toledo do COTP*.

Therefore, the intent is to identify, among the female athletes, knowledge about and the prevalence of the female athlete triad, of urinary incontinence, of eating disorders, of premenstrual tension, and of sexually transmitted diseases. It also seeks to identify the influence of the menstrual cycle on sports performance and on the relation between the athletes and their coaches as to gynecological issues, besides the identification of women without gynecological accompaniment so that they might receive early diagnosis and appropriate multidisciplinary follow-up when necessary.

These forms were evaluated by means of statistical analysis, using the Statistical Package for Social Sciences (SPSS) program, version v18.0. To compare qualitative variables, Fisher’s exact test was used. To compare continuous variables, variance analysis (ANOVA) was utilized, followed by Tukey’s *post-hoc* test. The data are presented as mean±standard deviation. A significance level of p=0.05 was adopted.

### Description of the proposed questionnaire

PPGE was prepared with 39 questions, as demonstrated in appendix 1.

This is the first tool with the proposal of a specific female evaluation within the national context, and has been used by the Brazilian Olympic Committee since 2011.

## RESULTS

The athletes had a mean age of 15.4±2.0 years and a mean body mass index (BMI) of 21.6 (±2.8) kg/m^2^, as is shown on table 1.

**Table 1:** Distribution of the demographic variables and training characteristics, as per sports modality

Modality	n	Age (years)mean±SD	BMI (kg/m^**2**^) mean±SD	Start of training (age in years) mean±SD	Training/week (hours) mean±SD
Track and field	21	14.8±2.0	19.9±3.3	12.6±1.4	14.2±4.9
Basketball	12	16.1±0.9	22.3±3.6	10.3±1.1	12.0±0.0
Boxing	4	14.5±1.9	24.6±4.8	14.3±1.7	9.1±3.3
Soccer	44	16.3±2.2	21.5±1.7	13.5±2.5	12.0±3.9
Handball	48	14.9±1.5	22.2±2.4	12.1±1.5	9.6±3.0
Judo	6	15.8±3.3	20.6±4.2	9.3±2.8	10.0±.0
Wrestling	7	16.0±1.3	23.3±2.9	15.0±1.2	8.6±2.9
Swimming	6	13.7±1.2	19.2±2.0	11.3±3.4	3.5±0.0

Total	148	15.4±2.0	21.6±2.8	12.6±2.3	10.9±4.0

BMI: body mass index; SD: standard deviation.

The table further demonstrates that the boxing athletes presented with a significantly higher BMI (24.6±4.8) than women of the track and field modality (19.9±3.3; p=0.041) and of the swimming modality (19.2±2.0; p=0.046). Additionally, on average, swimmers train for a significantly smaller number of hours (3.5±0.0 hours) than do participants in track and field (14.2±4.9 hours), basketball (12.0±0.0 hours), and soccer (12.0±3.9 hours) (p<0.0001).

The athletes evaluated initiated competitive training at an average age of 12.6±2.3 years and trained, on average, 10.9±4.0 hours/week, participating in the main regional and state tournaments of their categories (Table 1).

The mean age at menarche was 12.4±1.3 years, not differing significantly among the sports modalities (p=0.078), as shown on table 2.

**Table 2:** Data in reference to menstrual history as per sports modality

Modality	Menarche	Amenorrhea (%)	Irregular cycles (%)	Training during menstruation (%)
**(age in years)mean±SD**
Track and field	12.3±1.9	14.30	42.90	95.00
Basketball	13.2±1.3	0.00	45.50	100.00
Boxing	11.3±2.1	50.00	75.00	75.00
Soccer	12.7±0.9	11.90	62.50	95.20
Handball	12.0±1.3	6.50	41.30	97.80
Judo	12.4±1.5	66.70	20.00	100.00
Wrestling	11.7±1.1	28.60	71.40	100.00
Swimming	12.3±0.6	0.00	66.70	100.00

Total	12.4±1.3	13.50	50.40	96.40

As to regularity of the menstrual cycle, 50.4% of the total sample reported having irregular cycles (non-monthly or undefined intervals), with reports of 13.5% of amenorrhea (absence of menstruation for 3 or more months) (Table 2). No statistically significant differences were observed among the modalities as to menstrual irregularity (p=0.272). On the other hand, reports of amenorrhea proved significantly more frequent in athletes from sports, such as judo (66.7%) and boxing (50%), when compared to basketball (0%), handball (6.5%), swimming (0%), wrestling (28.6%), and track and field (14.3%) (p=0.003).

Among those evaluated, 96.4% reported training habitually during their menstrual period, with no significant differences among the modalities (p=0.497).

**Figure 1:**
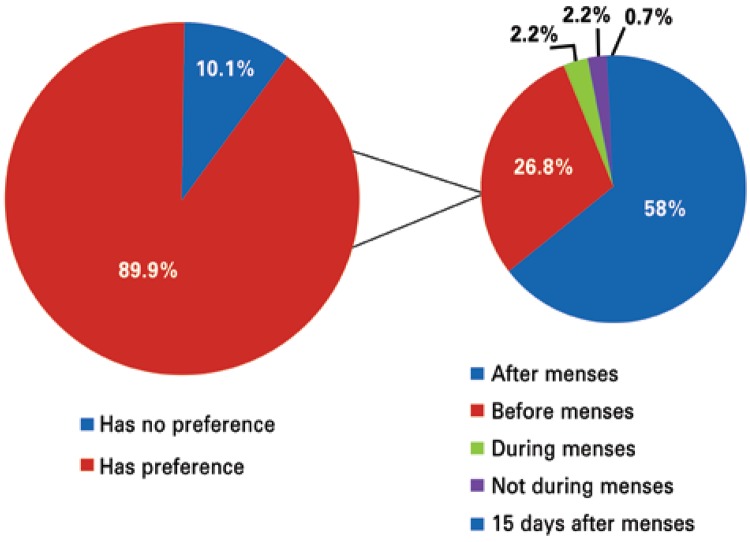
Period of the menstrual cycle in which the athlete prefers to compete

As to sports performance, 89.9% of the athletes reported preferring a certain phase of the cycle for competing. Among them, 58.0% preferred the postmenstrual period, such as is shown on figure 1.

**Figure 2:**
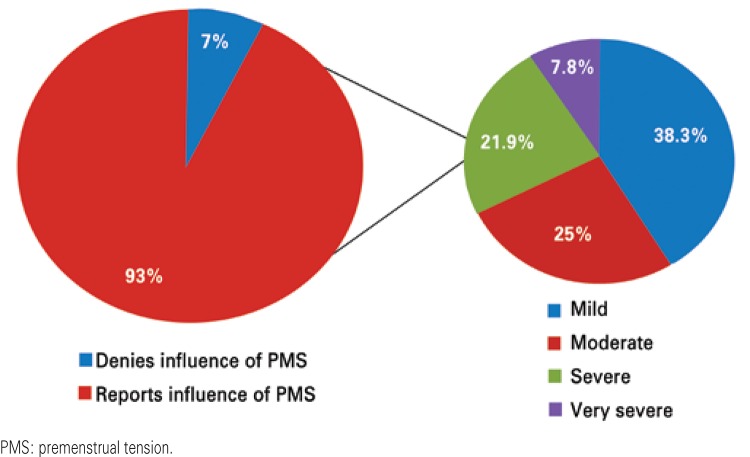
Influence of the premenstrual symptoms on sports performance

In the total sample, only 7% of the athletes denied the influence of PMS symptoms; 38.3% reported experiencing light symptoms; 25%, moderate symptoms; 21.9%, severe; and 7.8%, very severe symptoms, as can be seen in figure 2.

As to sex life, 17.7% of the total number of women evaluated reported having sexual relations, in which 92.4% denied using contraceptive measures and 23% of the athletes had no knowledge of STDs. Such lack of knowledge is significantly greater in women of the swimming and judo modalities (50%), when compared to those of basketball (0%) and handball (10.9%) (p=0.005).

Additionally, among these women, 72.4% denied having gynecological accompaniment at least once a year, as is presented on table 3.

**Table 3:** Distribution of the sexual variables as per sports modalities

Modality	Coitarche Yes (%)	Contraception methods Yes (%)	Knowledge about sexually transmitted diseases No (%)	Gynecological accompaniment No (%)
Track and field	19.00	5.00	20.00	80.00
Basketball	8.30	50.00	0	50.00
Boxing	0	0	0	100.00
Soccer	20.50	2.60	39.00	75.00
Handball	16.70	16.70	10.90	70.20
Judo	16.70	0	50.00	50.00
Wrestling	50.00	0	16.70	71.40
Swimming	0	0	50.00	100.00

Total	17.70	7.60	23.00	72.40

Patients with an active sexual life denied the occurrence of prior gestations, and therefore, all were nulligravida.

Among the athletes evaluated, 89.9% reported never having heard about the female athlete triad, with no significant differences among the sports (p=0.699).

As to bone alterations, reports were obtained of 5.4% stress fractures, as demonstrated on table 4, with no statistical difference among the modalities (p=0.632).

**Table 4:** Distribution of stress fracture reports and knowledge of the female athlete triad and urinary incontinence, per sports modality

Modality	Stress fractures Yes (%)	Knowledge about female athlete triad Yes (%)	Knowledge about urinary incontinence Yes (%)
Track and field	0	4.80	14.30
Basketball	8.30	8.30	8.30
Boxing	0	25.00	25.00
Soccer	4.70	9.10	11.60
Handball	8.30	14.60	6.40
Judo	16.70	16.70	33.30
Wrestling	0	0	0
Swimming	0	0	16.70

Total	5.40	10.10	11.00

Surgical findings included, especially, orthopedic and otorhinolaryngologic (ENT) operations, with no gynecological operations that might interfere in the symptoms investigated.

From the results obtained in the EAT-26 analysis, 9.5% of the athletes obtained scores above 20 and were, therefore, considered at greater risk for the development of eating disorders. Additionally, of the total number of athletes assessed, 5.5% reported using laxatives, diuretics, or inducing vomiting to maintain weight.

None of the athletes reported the three symptoms of the triad, characterized in this study by concomitant EAT-26≥20, menstrual irregularity or amenorrhea, and stress fractures. However, 59.4% reported at least one of the three symptoms, and 5.1% presented with two of them. The association among the symptoms, by modality, was not statistically different (p=0.095).

As to urinary symptoms, 89% of the athletes were not aware of the fact that, despite not presenting with the classic risk factors, they could be at an increased risk of urinary incontinence. There was no statistically significant difference among the modalities (p=0.351) (Table 4).

Among the athletes assessed, 18.2% reported some degree of urine loss, with 4.9% reaching scores >8. The difference, however, was not statistically significant among the different sports (p=0.833).

## DISCUSSION

The study was conducted with a young population, with a mean de age of 15.4 years, which could be considered adolescents. We need to point out the importance of dissemination of information at this age, considering the psychological, physical, and social changes the adolescents are exposed to.

Among the variables studied, the age at menarche was 12.4±1.3 years, similar to that of the general Brazilian population, which is 12.6 years.^(11)^ We emphasize the concern with the analysis of this fact since the practice of competitive sports activity, generally associated with more intense training, is related to delayed pubertal development and consequently, of menarche.^(12)^

The evaluation that 72.4% of the girls denied having gynecological accompaniment, 23% did not know the meaning of STDs (sexually transmitted diseases), as well as 92.4% of those interviewed reporting having an active sex life without the use of contraceptives, demonstrates that the lack of information, the risk of an undesired pregnancy, and the lack of care specifically related to gynecological issues may place at risk the health of these athletes and consequently, determine the sudden interruption of their sports life.

As to the female athlete triad, the occurrence of urinary incontinence and the possibility of planning the menstrual cycle relative to the sports competition calendar to minimize possible losses due to PMS symptoms, we noted that these girls do not know or they underestimate the risks inherent to their behaviors. Our data confirm this information in demonstrating that 89.9% are unaware of the triad, despite 59.4% of them presenting with a symptom related to it, and 5.1% presenting with two of them.

As to urinary incontinence, 89% are not aware of the possibility of involuntary urine loss in the population of athletes, despite the fact that 18.2% reported some degree of loss, with 4.9% having a score >8, which characterizes possible hindrance to quality of life.

Regarding PMS, we verified that almost all athletes (93%) reported symptoms associated with the syndrome, which are treatable, with likely improvement of quality of life and of sports performance. It is important to point out that these symptoms should be valued, since they especially affect women between the second and third decades of life. However, when diagnosed, these women reported about 10 years of complaints,^(13)^ which lead us to conclude that the symptoms may initiate while they are yet in adolescence, the age of this study population.

Despite the gold medals having been obtained at the different phases of the menstrual cycle throughout their history,^(14)^ planning of the menstrual cycle by women through hormonal contraceptives is a modern conquest. In the case of the athletes, we can allow them to choose the phase the cycle when they feel best prepared to compete, whether based on physical tests or on subjective reports. We noted that 89.9% of those evaluated would choose one phase of the cycle if they had the option.

We believe in the importance of guidance for the athletes, as well as for all professionals involved in their care. Fitness instructors and coaches, in recognizing and valuing the hormonal variations that their athletes are susceptible to and the risks they are exposed to when not duly oriented, can guarantee the health of their athletes and possibly, better sports results.

We point out that it may be necessary that the athletes, when also informed about the possible benefits, will feel more comfortable in discussing this topic with their coaches. To be open to information and to increasing one’s knowledge about the particularities one is exposed to are indispensable factors when training women.^(15)^

Once any possible alterations are identified, the athletes should be referred to professionals who can help them and accompany them. In this study, the acknowledgment of the athletes as to urine loss (23.1%) caused them to be referred to evaluations by a physical therapist specialized in urogynecology, and those at greater risk of eating disorders, diagnosed by means of the EAT-26 (9.5%), or those who reported the use of laxatives or diuretics to maintain or lose weight (4.5%), were referred to a detailed dietary assessment and follow-up, if necessary. In our studies, the greatest averages in score were found in the sports that require rigid weight control, such as judo, boxing, and wrestling.

Additionally, all were offered gynecological accompaniment at the same medical department of COTP. At this site, they could undergo individualized gynecological consultations in order to solve remaining questions related to the issues presented in the lectures, or be treated for preexisting complaints.

Information and recognition of alert signals are still the most important measures to be taken. Troy et al. demonstrated that only 9% of the physicians felt comfortable treating athletes with the triad,^(16)^ which warns us as to the need to disseminate this information among the professionals involved in treating these women.

The lack of adequate protocols is still the greatest difficulty and the greatest criticism for the studies that seek to evaluate the prevalence of gynecological alterations and of the female athlete triad among sportswomen. The other particularities, such as urinary incontinence, PMS, and the influence of the menstrual cycle on sports performance, however, are also susceptible to the same criticism and to the lack of definite explanations, which are usually not valued. This fact should, therefore, confirm the importance of active investigation and of early intervention that might guide and guarantee the health of these girls.

This study did not have the objective of effectively comparing statistics among modalities, since the number of participants in each group was not uniform. The analyses were, however, performed and mentioned when pertinent.

This study reiterated the fact that the gynecological issues and particularities of the female athlete should be actively investigated and not underestimated during her accompaniment, since they are relevant to her health, and consequently, to her sports performance.

## CONCLUSION

The tool we propose, called Pre-Participation Gynecological Examination, allowed the identification of issues that are generally not reported during the conventional pre-participation evaluation.

Specific care of the female athlete, often underestimated, proved to be of extreme importance and the tool we propose would be of great value for tracking the particular alterations of this population. The Pre-Participation Gynecological Examination may be applied by the physician in charge during the female pre-participation evaluation, identifying alterations and thus allowing referrals to specialized professionals.

## Appendix 1

**Table:** Pre-Participation Gynecological Examination (PPGE)

General data
1) Date of birth (D/M/Y): __________Weight_______ Height______
Race (_) White__(_) Non-white
2) Level of schooling
(_) Elementary School (1st – 8th grade)__(_) Middle/High School
(_) Higher education__(_) Complete__(_) Incomplete course:_____________________
**Sports history**
3) Modality: ____________
4) Age at start of competitive training: _______ years
5) Hours of training per day, currently: ______ hours
6) Weekly frequency of practice, currently:__________ days/week
**Menstrual antecedents **
7) Age at first menstruation: ______ years
8) Do you practice while menstruated? (_) Yes__(_) No
9) How many days does your menstruation last? _____ days
10) How long afterwards do you menstruate again?
(_) I don’t know because it varies a lot
(_) I don’t know, but I menstruate once a month
(_) After ____ days
11) Have you gone 3 months (or more) without menstruating? (_) Yes__(_) No
12) If yes: was it during a period of intensive training? (_) Yes__(_) No
Were you lower than your normal weight? (_) Yes__(_) No
Did your period normalize once the rhythm of training decreased? (_) Yes__(_) No
Did it normalize when you gained weight? (_) Yes__(_) No
It is not associated with training or change in weight (_)
**Influence of menstrual cycle on performance**
13) Does your coach know when you are in your menstrual period? (_) Yes__(_) No
14) Does your coach agree to change your training if menstruation is interfering with your performance? (_) Yes__(_) No__(_) I don’t tell the coach or talk to him/her about it
15) If you could choose a time to compete, you would choose:
(_) My menstrual period does not influence me__(_) Not during menstruation
(_) Right before menstruation__(_) During menstruation
(_) Right after menstruation__(_) More or less 15 days after menstruation
**PMS**
16) What do you feel before your menstrual period? Attribute a score: 1 (if it does not bother you) or 2 (if it bothers you a lot).
(_) I do not feel anything that really bothers me __
(_) Pain or swelling in the breasts __
(_) Swelling in my belly, feeling of weight or discomfort __
(_) Headache __
(_) Swelling in hand or legs __
(_) Increase in body weight __
(_) Pain the back, joints, or muscles __
(_) Irritation, nervousness, or sadness __
(_) Increase in appetite __
(_) Changes in sleep __
(_) Lack of concentration, loss of motivation __
(_) Menstrual cramps __
**Sexual history**
17) Have you had sexual relations with men? (_) Yes__(_) No
18) If yes, how old were you “in the first time”? _______ years
19) How many partners have you had? _______
20) Do you have a fixed partner (you have intercourse only with this person)? (_) Yes__(_) No
21) If “yes”, for how long? __________
22) Have you had sexual relations with women? (_) Yes__(_) No
23) Do you use any contraceptive method? (_) Yes__(_) No
24) If “yes”, mark the correct option:
(_) Pill:__________(_) IUD with progesterone__(_) Male condom
(_) Injection once a month__(_) Copper IUD__(_) Female condom
(_) Injection every 3 months__(_) Vaginal ring__(_) Other_______________
25) Was it prescribed by a gynecologist? (_) Yes__(_) No
26) Started to use it to: (_) Avoid pregnancy__(_) Regulate menstruation__(_) For both reasons
27) Do you know what a STD is? (_) Yes__(_) No
28) Have you ever had to treat any sexually transmitted disease? (_) Yes__(_) No
29) If “yes”:
Which disease? ___________________________________
What was the treatment? ________________________________
Were you cured? (_) Yes__(_) No
**Obstetric history**
30) Have you ever been pregnant?: Yes (_)__No (_)__How many times? __________
Number of deliveries (write the number): (_) Normal__(_) C-section__(_) Forceps
Number of abortions (write the number): (_) Spontaneous/Miscarriage__(_) Provoked
**Female athlete triad**
31) Have you ever heard of the “female athlete triad”? Yes (_)__No (_) __________
32) Have you ever had a stress fracture? (A fracture not associated with trauma, accident, or acute torsion) (_) Yes (_) No
33) If “yes”, at that time:
Were you menstruating normally? (_) Yes__(_) No__(_) I don’t remember
Had you lost weight? (_) Yes _______kg__(_) No__(_) I don’t remember
Were you at a time of intense training? (_) Yes__(_) No__(_) I don’t remember
In which region of the body did it occur? __________________________
**Past medical/surgical history **
34) Are you under treatment for any disease? (_) Yes__(_) No.__Which ?_________________________
35) Do you regularly take any medication? (_) Yes__(_) No.__Which?_______________
36) Have you had any operations? (_) Yes__(_) No.__Which?_____________When? ________
37) Are you allergic to any food or medication? (_) Yes__(_) No
38) If “yes”, to what? _______________________________________
39) Do you have routine gynecological accompaniment (at least once a year)? (_) Yes__(_) No
