# Supplementary material for: Pre-participation gynecological evaluation of female athletes: a new proposal
Source: Einstein (Sao Paulo). 2014 Oct-Dec;12(4):459–66. doi: 10.1590/S1679-45082014AO3205 (PMC4879912; doi:10.1590/S1679-45082014AO3205)
Supplement: Translation

# Translation: Avaliação ginecológica pré-participação da mulher atleta: uma nova proposta

**Authors:** Tathiana Rebizzi Parmigiano, Eliana Viana Monteiro Zucchi, Maíta Poli de Araujo, Camila Santa Cruz Guindalini, Rodrigo de Aquino Castro, Zsuzsanna Ilona Katalin de Jármy Di Bella, Manoel João Batista Castello Girão, Moisés Cohen, Marair Gracio Ferreira Sartori

**Affiliations:** 1 Universidade Federal de São Paulo, São Paulo, SP, Brasil.

**Keywords:** Síndrome da tríade da mulher atleta, Incontinência urinária por estresse, Síndrome pré-menstrual, Desempenho atlético, Questionários

## Abstract

**Objetivo:**

Propor a inclusão da investigação ginecológica durante a avaliação pré-participação de mulheres praticantes de exercício físico, por meio de um instrumento específico, denominado *Pre-Participation Gynecological Examination (PPGE)*.

**Métodos:**

Foram avaliadas 148 atletas com média de idade de 15,4±2,0 anos, participantes de oito diferentes modalidades esportivas, que responderam ao *Pre-* *Participation Gynecological Examination*, ao *International Consultation on Incontinence Questionnaire - Short Form* (para avaliar perda urinária) e ao *Eating Attitudes Test *(para avaliar alterações alimentares).

**Resultados:**

Cinquenta por cento das atletas referiram irregularidade menstrual, 23% desconheciam as doenças sexualmente transmissíveis e 72,4% negaram acompanhamento ginecológico, no mínimo, anual. Foram identificados 18,2% de mulheres com perda urinária e 15% de maior risco de alterações alimentares. Além disso, 89,9% desconheciam a existência de incontinência urinária em atletas ou o fato de estarem suscetíveis à tríade da mulher atleta. Entre elas, ainda, 87,1% afirmaram não se referirem a seus técnicos sobre as questões ginecológicas, mesmo que isso pudesse melhorar sua saúde e seu desempenho.

**Conclusão:**

O questionário *Pre-Participation Gynecological Examination* mostrou-se um instrumento de fácil aplicabilidade e permitiu o diagnóstico de alterações por vezes não valorizadas pelas próprias atletas. Após a aplicação do questionário, as alterações puderam ser identificadas, determinando o encaminhamento das atletas para avaliação e tratamento adequados.

## INTRODUÇÃO

A vida sedentária está relacionada a uma quantidade imensa de riscos à saúde. Em contraste, pessoas expostas a um estilo de vida extremamente ativo ou atletas em nível competitivo também estão suscetíveis a um número importante de ameaças a saúde e ao bem-estar, principalmente na ausência de acompanhamento médico adequado.^(1)^

Sabe-se que algumas alterações podem ser preexistentes e outras exacerbadas durante a prática esportiva. Por essa razão, propõe-se a realização de uma avaliação pré-participação a todos os indivíduos dispostos a iniciar ou a manter uma rotina de exercício físico − avaliação esta denominada *Pre-Participation Examination* (PPE). O objetivo da PPE é promover a saúde e a segurança dos esportistas em treinamentos e competições, e não excluí-los das atividades.^(2)^

Neste contexto, apesar de desde 2007 o *American College of Sports Medicine* (ACSM) ter preconizado a importância da PPE especificamente em mulheres atletas, essa prática ainda não é comum, principalmente em nosso país. A PPE não está implementada adequadamente ou uniformemente, por isso são grandes os esforços no estabelecimento de protocolos.^(3)^ Foi apenas em 2010 que, pela primeira vez, o cuidado específico com a mulher atleta foi oficialmente proposto, por meio da investigação de questões que podem afetar sua saúde e sua participação em atividades esportivas.^(4)^

A avaliação de algumas características do ciclo menstrual, como a ocorrência e a idade da menarca, data de última consulta ginecológica, presença de amenorreia por mais de 6 meses e o reconhecimento da relação entre menstruação e desempenho físico, há tempo é motivo de estudo. Ressalta-se que a PPE pode ser, em alguns casos, o único contato da atleta com um médico, aumentando ainda mais a importância dessa avaliação.^(5)^

Com o aumento da participação feminina nos esportes, é imprescindível que algumas questões, como a tríade da mulher atleta, a tensão pré-menstrual (TPM) e a incontinência urinária, sejam informadas às mulheres esportistas, sejam estas de elite ou não. Muitas mulheres, assim como os profissionais que cuidam de seu bem-estar e de seus treinos, estão desavisadas das alterações às quais estão suscetíveis e desconhecem que estão colocando em risco sua saúde e, em segundo plano, seu rendimento esportivo.^(6)^

Rumball e Lebrum.^(6)^ sugeriram a necessidade de se reservar uma parte específica da avaliação para as questões inerentes ao organismo feminino, com investigações ginecológicas e nutricionais específicas e direcionadas, evitando que esses temas sejam omitidos por essas mulheres ou que seus cuidados sejam subestimados pelos profissionais envolvidos. Isso condiz com estudo publicado por Careck e Futrell, que ressaltaram que as mulheres não se sentem confortáveis em responder sobre questões que envolvam aspectos ginecológicos, uso de álcool e drogas, e hábitos alimentares na forma tradicional de avaliação.^(7)^

No Brasil como no resto do mundo, a Sociedade Brasileira de Medicina do Esporte também reconhece as diferenças metabólicas entre homens e mulheres, e posiciona-se em relação às particularidades da mulher que pratica atividade física sem, entretanto, apresentar nenhum protocolo específico, até o momento, para esse cuidado.^(8)^

Questões ginecológicas não fazem parte da avaliação pré-participação convencional, caracterizada exclusivamente pela abordagem de aspectos ortopédicos e cardiológicos. O instrumento apresentado é o primeiro em contexto nacional que valoriza essa investigação específica e que propõe sua inclusão durante esse momento de avaliação.

## OBJETIVO

Propor a inclusão da investigação ginecológica durante a avaliação pré-participação de mulheres praticantes de exercício físico, por meio de um instrumento específico, denominado *Pre-Participation Gynecological Examination *(PPGE).

## MÉTODOS

O estudo foi realizado no centro esportivo Centro Olímpico de Treinamento e Pesquisa (COTP), localizado em São Paulo (SP), durante o primeiro semestre de 2010. Trata-se de um local reconhecido como formador de atletas de dez diferentes modalidades esportivas, sendo três exclusivamente femininas (futebol, handebol e basquete), seis mistas (luta olímpica, judô, atletismo, ginástica artística, natação e boxe) e uma masculina (voleibol). Sua equipe médica foi a primeira no país a incluir em sua equipe uma ginecologista especializada no atendimento de mulheres atletas.

O projeto foi aprovado pelo Comitê de Ética da Escola Paulista de Medicina da Universidade Federal de São Paulo, sob o título de “Avaliação ginecológica da mulher atleta”, sob a inscrição 1.772/10 neste comitê e pela Coordenadoria de Gestão do Esporte de Alto Rendimento do COTP.

Foi proposta, mediante preenchimento de Termo de Consentimento Livre e Esclarecido pelo responsável, a realização de palestras informativas relacionadas à saúde feminina e a aspectos relacionados à mulher esportista, como anatomia feminina, fisiologia do ciclo menstrual, métodos contraceptivos, doenças sexualmente transmissíveis (DSTs), exame preventivo para câncer de colo do útero, tríade da mulher atleta, incontinência urinária em atletas, TPM, e relação entre contraceptivos e *doping*. Foram critérios de inclusão para que participassem das palestras: idade acima de 13 anos, consentimento dos pais devidamente assinado e estarem em período compatível com a renovação anual da avaliação pré-participação ou sua primeira realização.

Participaram das palestras 148 atletas com média de idade de 15,4±2,0 anos integrantes das oito diferentes modalidades esportivas que apresentam praticantes mulheres do COTP.

Após as palestras, foram distribuídos os questionários a cada atleta para a obtenção de informações individuais referentes ao histórico ginecológico. Esse questionário foi denominado “*Pre-Participation Gynecological Examination*”. O modelo proposto foi formulado pela primeira autora deste estudo, é autoaplicável e não depende de supervisão para seu preenchimento (Anexo 1).

Ao final, foram anexados para respostas o questionário *International Consultation on Incontinence Questionnaire-Short Form* (ICIQ-SF), validado e traduzido para a língua portuguesa, para a avaliação de perda involuntária de urina,^(9) ^e o *Eating Attitudes Test* ^*®*^ (EAT-26), para alterações alimentares.^(10)^ Posteriormente, todas as informações obtidas foram anexadas aos prontuários médicos do Departamento Médico do Centro de Excelência em Medicina Esportiva Caio Pompeu de Toledo do COTP.

Dessa maneira, visa-se identificar entre as mesmas o conhecimento e a prevalência da tríade da mulher atleta, de incontinência urinária, de alterações alimentares, de tensão pré-menstrual e de doenças sexualmente transmissíveis. Visa, ainda, identificar a influência do ciclo menstrual no desempenho esportivo e no relacionamento das atletas com seus técnicos, no que diz respeito às questões ginecológicas, além de buscar, nessa população, mulheres sem acompanhamento ginecológico, para que se possam propiciar diagnóstico precoce e acompanhamento multidisciplinar adequado, quando necessário.

Esses formulários foram avaliados por meio de análise estatística, utilizando o pacote estatístico *Statistical Package for Social Sciences* (SPSS), versão v18.0. Para comparação de variáveis qualitativas, foi utilizado o teste exato de Fisher. Para comparação das variáveis contínuas, foi utilizado o teste de análise de variância (ANOVA) seguido do teste *post-hoc* de Tukey. Os dados estão representados como média±desvio padrão. O nível de significância foi p=0,05.

### Descrição do questionário proposto

O PPGE foi elaborado com 39 questões, conforme demonstrado no anexo 1.

Trata-se do primeiro instrumento com a proposta de avaliação específica feminina em contexto nacional, e tem sido usado pelo Comitê Olímpico Brasileiro desde 2011.

## RESULTADOS

As atletas apresentaram média de idade de 15,4±2,0 anos e média de índice de massa corporal (IMC) de 21,6 (±2,8) kg/m^2 ^como demonstrado na tabela 1.

**Tabela 1:** Distribuição das variáveis demográficas e características de treinamento, de acordo com a modalidade esportiva

Modalidade	n	Idade (anos) média±DP	IMC (kg/m^2^) média±DP	Início do treinamento (idade em anos) média±DP	Treino/semana (horas) média±DP
Atletismo	21	14,8±2,0	19,9±3,3	12,6±1,4	14,2±4,9
Basquete	12	16,1±0,9	22,3±3,6	10,3±1,1	12,0±0,0
Boxe	4	14,5±1,9	24,6±4,8	14,3±1,7	9,1±3,3
Futebol	44	16,3±2,2	21,5±1,7	13,5±2,5	12,0±3,9
Handebol	48	14,9±1,5	22,2±2,4	12,1±1,5	9,6±3,0
Judô	6	15,8±3,3	20,6±4,2	9,3±2,8	10,0±,0
Luta olímpica	7	16,0±1,3	23,3±2,9	15,0±1,2	8,6±2,9
Natação	6	13,7±1,2	19,2±2,0	11,3±3,4	3,5±0,0

Total	148	15,4±2,0	21,6±2,8	12,6±2,3	10,9±4,0

IMC: índice de massa corporal; DP: desvio padrão.

A tabela demonstra ainda que as atletas da modalidade boxe apresentam IMC significativamente maior (24,6±4,8) do que mulheres da modalidade atletismo (19,9±3,3; p=0,041) e da modalidade natação (19,2±2,0; p=0,046). Além disso, nadadoras treinam em média uma quantidade de horas significativamente menor (3,5±0,0 horas) do que praticantes do atletismo (14,2±4,9 horas), basquete (12,0±0,0 horas) e futebol (12,0±3,9 horas) (p<0,0001).

As atletas avaliadas iniciaram treinamento competitivo com média de 12,6±2,3 anos e treinavam, em média, 10,9±4,0 horas/semana, participando dos principais campeonatos regionais e estaduais de suas categorias (Tabela 1).

A média da idade da menarca foi de 12,4±1,3 anos, não diferindo significativamente entre as modalidades esportivas (p=0,078), como demonstrado na tabela 2.

**Tabela 2:** Dados referentes aos antecedentes menstruais conforme modalidade esportiva

Modalidade	Menarca	Amenorreia (%)	Ciclos irregulares (%)	Treinamento menstruada (%)
	(idade em anos) Média±DP			
Atletismo	12,3±1,9	14,30	42,90	95,00
Basquete	13,2±1,3	0,00	45,50	100,00
Boxe	11,3±2,1	50,00	75,00	75,00
Futebol	12,7±0,9	11,90	62,50	95,20
Handebol	12,0±1,3	6,50	41,30	97,80
Judô	12,4±1,5	66,70	20,00	100,00
Luta olímpica	11,7±1,1	28,60	71,40	100,00
Natação	12,3±0,6	0,00	66,70	100,00

Total	12,4±1,3	13,50	50,40	96,40

Em relação à regularidade do ciclo, 50,4% do total da amostra referiu ciclos irregulares (intervalos não mensais ou indefinidos), com relato de 13,5% de amenorreia (ausência de menstruação por 3 ou mais meses) (Tabela 2). Não foram observadas diferenças estatisticamente significantes entre as modalidades em relação à irregularidade menstrual (p=0,272). Por outro lado, os relatos de amenorreia mostraram-se significativamente mais frequentes em atletas de esportes como judô (66,7%) e boxe (50%), quando comparados ao basquete (0%), ao handball (6,5%), à natação (0%), à luta olímpica (28,6%) e ao atletismo (14,3%) (p=0,003).

Entre as avaliadas, 96,4% referiam treinar habitualmente durante o período menstrual, não havendo diferenças significantes entre as modalidades (p=0,497).

Em relação ao desempenho esportivo, 89,9% das atletas referiram preferir uma determinada fase do ciclo para competirem. Entre estas, 58,0% preferiram o período pós-menstrual, como demonstrado na figura 1.

**Figura 1:** Período do ciclo menstrual em que a atleta prefere competir

Além disso, 87,1% do total das atletas referiu não comentar com seus técnicos sobre seu fluxo menstrual e 81,9% acreditavam ainda que os treinos não seriam alterados devido aos sintomas relacionados ao seu ciclo menstrual.

Na amostra total, apenas 7% das atletas negavam influência dos sintomas de TPM; 38,3% referiram sintomas leves; 25%, sintomas moderados; 21,9%, graves; e 7,8%, sintomas muito graves, conforme pode ser verificado na figura 2.

**Figura 2:** Influência dos sintomas de tensão pré-menstrual sobre o desempenho esportivo

Em relação à vida sexual, 17,7% do total de mulheres avaliadas referiram relação sexual, sendo que 92,4% negaram uso de métodos anticoncepcionais e 23% das atletas não tinham conhecimentos sobre DST. Tal falta de conhecimento é significativamente maior em mulheres das modalidades natação e judô (50%), quando comparadas as das modalidades basquete (0%) e handebol (10,9%) (p=0,005).

Além disso, entre essas meninas, 72,4% negaram acompanhamento ginecológico, no mínimo uma vez ao ano, como apresentado na tabela 3.

**Tabela 3:** Distribuição das variáveis sexuais de acordo com as modalidades esportivas

Modalidade	Coitarca Sim (%)	Métodos anticoncepcionais Sim (%)	Conhecimento sobre doenças sexualmente transmissíveis Não (%)	Acompanhamento ginecológico Não (%)
Atletismo	19,00	5,00	20,00	80,00
Basquete	8,30	50,00	0	50,00
Boxe	0	0	0	100,00
Futebol	20,50	2,60	39,00	75,00
Handebol	16,70	16,70	10,90	70,20
Judô	16,70	0	50,00	50,00
Luta olímpica	50,00	0	16,70	71,40
Natação	0	0	50,00	100,00

Total	17,70	7,60	23,00	72,40

As pacientes com vida sexual ativa negaram a ocorrência de gestação anterior, sendo, desse modo, todas nuligestas.

Entre as atletas avaliadas, 89,9% relataram que nunca haviam ouvido falar sobre a tríade da mulher atleta, sem diferenças significativas entre os esportes (p=0,699).

Em relação a alterações ósseas, foram obtidos relatos de 5,4% de fraturas por estresse, conforme demonstrado na tabela 4, sem diferença estatística entre as modalidades (p=0,632).

**Tabela 4:** Distribuição de relato de fratura por estresse e conhecimento sobre a tríade da mulher atleta e incontinência urinária, por modalidade esportiva

Modalidade	Fraturas de estresse Sim (%)	Conhecimento sobre a tríade da mulher atleta Sim (%)	Conhecimento sobre incontinência urinária
			Sim (%)
Atletismo	0	4,80	14,30
Basquete	8,30	8,30	8,30
Boxe	0	25,00	25,00
Futebol	4,70	9,10	11,60
Handebol	8,30	14,60	6,40
Judô	16,70	16,70	33,30
Luta olímpica	0	0	0
Natação	0	0	16,70

Total	5,40	10,10	11,00

Os achados cirúrgicos incluíam, sobretudo, cirurgias ortopédicas e otorrinolaringológicas, não havendo a ocorrência de cirurgias ginecológicas que pudessem interferir nos sintomas investigados.

A partir dos resultados obtidos da análise do EAT-26, 9,5% das atletas obtiveram pontuação acima de 20, e, assim, foram consideradas com maior risco para o desenvolvimento de alterações alimentares. Além disso, do total de atletas avaliadas, 5,5% referiram o uso de laxantes, diuréticos ou indução de vômito para a manutenção de peso.

Nenhuma das atletas referiu os três sintomas da tríade, caracterizados neste estudo por EAT-26≥20, irregularidade menstrual ou amenorreia, e fraturas por estresse, concomitantemente. Entretanto, 59,4% relataram ao menos um entre os três sintomas e 5,1% apresentavam dois deles. A associação entre os sintomas, por modalidade, não foi estatisticamente diferente (p=0,095).

Em relação aos sintomas urinários, 89% das atletas desconheciam o fato de que, apesar de não apresentarem os clássicos fatores de risco, poderiam apresentar risco aumentado de incontinência urinária. Não houve diferença estatisticamente significante entre as modalidades (p=0,351) (Tabela 4).

Entre as atletas avaliadas, 18,2% relataram algum grau de perda urinária com 4,9% atingindo pontuações >8. A diferença, entretanto, não foi estatisticamente significativa entre os diferentes esportes (p=0,833).

## DISCUSSÃO

O estudo foi conduzido com uma população jovem, com média de idade de 15,4 anos, considerada, assim, de adolescentes. É preciso ressaltar a importância da disseminação da informação nessa idade, frente às várias alterações psicológicas, físicas e sociais às quais as adolescentes estão expostas.

Entre as variáveis estudadas, a idade de menarca foi de 12,4±1,3 anos, semelhante a da população geral brasileira, que é de 12,6 anos.^(11)^ Ressaltamos a preocupação com a análise desse dado pois a prática de atividade esportiva competitiva, geralmente associada a treinamentos mais intensos, está relacionada ao atraso do desenvolvimento puberal e, consequentemente, da menarca.^(12)^

A avaliação de que 72,4% das meninas negavam acompanhamento ginecológico, 23% desconheciam ao menos o significado de DSTs (doenças sexualmente transmissíveis), assim como 92,4% das entrevistadas com vida sexual ativa não utilizavam qualquer contraceptivo, demonstra que a falta de informação, o risco de uma gestação indesejada e a falta de cuidado especificamente relacionado às questões ginecológicas podem colocar em risco a saúde dessas atletas e, consequentemente, determinar a interrupção súbita da vida esportiva.

Em relação à tríade da mulher atleta, à ocorrência de incontinência urinária e à possibilidade de planejamento do ciclo menstrual em relação ao calendário competitivo para minimizar possíveis prejuízos impostos por sintomas de TPM, verificamos que essas mulheres desconhecem ou subestimam os riscos inerentes a seus comportamentos. Nossos dados comprovam essa informação ao demonstrar que 89,9% das atletas desconheciam a tríade, apesar de 59,4% delas apresentarem um sintoma relacionado a mesma e 5,1% apresentarem dois deles.

Em relação à incontinência urinária, 89% desconheciam a possibilidade de perda involuntária de urina entre a população de atletas, apesar de 18,2% terem referido algum grau de perda, com 4,9% apresentando pontuação >8, caracterizando possível prejuízo de qualidade de vida.

Em relação à TPM, verificamos que quase a totalidade das atletas (93%) referiram sintomas associados à síndrome, sendo estes passíveis de tratamento, com provável melhora da qualidade de vida e do rendimento esportivo. Vale ressaltar que esses sintomas devem ser valorizados, por acometerem principalmente mulheres entre as segunda e terceira décadas de vida. Entretanto, quando diagnosticadas, essas mulheres relataram cerca de 10 anos de queixas,^(13)^ o que nos leva a concluir que os sintomas podem se iniciar ainda na adolescência, idade da população deste estudo.

Apesar de medalhas de ouro terem sido obtidas nas diferentes fases do ciclo ao longo da história,^(14)^ o planejamento do ciclo menstrual pelas mulheres por meio do uso de contraceptivos hormonais é uma conquista atual. No caso das atletas, podemos permitir que elas escolham a fase do ciclo em que se sintam mais aptas para competir, seja isso baseado em testes físicos ou no relato subjetivo. Notamos que 89,9% das avaliadas escolheriam uma fase do ciclo, se lhes fosse dada a opção.

Acreditamos na importância da orientação das atletas, assim como de todos os profissionais envolvidos no cuidado das mesmas. Preparadores físicos e técnicos, ao reconhecerem e valorizarem as variações hormonais a que suas atletas estão suscetíveis e os riscos a que estão expostas diante da falta de orientação adequada, conseguem garantir a saúde das mesmas e, possivelmente, melhores resultados esportivos.

Ressaltamos que talvez seja necessário que as atletas, ao também serem informadas sobre os possíveis benefícios, sintam-se mais confortáveis em discutirem esse assunto com seus técnicos. Estar acessível à informação e aumentar seu conhecimento sobre as particularidades a que estão expostas são fatores imprescindíveis, ao se treinarem mulheres.^(15)^

Uma vez identificadas quaisquer das possíveis alterações, as atletas devem ser encaminhadas a profissionais que possam ajudá-las e acompanhá-las. Neste estudo, o reconhecimento das atletas com queixas de perda urinária (23,1%) fez com que elas fossem encaminhadas para avaliação por fisioterapeuta especializado em uroginecologia e, aquelas com maior risco de alterações alimentares, diagnosticadas por meio do EAT-26 (9,5%), ou ainda as que relataram o uso de laxantes ou diuréticos para manutenção ou perda de peso (4,5%), foram encaminhadas à avaliação nutricional minuciosa e ao acompanhamento, se necessário. Em nossos estudos, as maiores médias de pontuações foram encontradas nos esportes cujas categorias exigem controle de peso, como o judô, boxe e luta olímpica.

Além disso, a todas foi oferecido acompanhamento ginecológico no próprio departamento médico do COTP. Nesse local, elas poderiam passar por consulta ginecológica individualizada, para, dessa maneira, solucionarem dúvidas remanescentes às questões apresentadas na palestra ou serem atendidas por queixas já existentes.

A informação e o reconhecimento de sinais de alerta são, ainda, as medidas mais importantes a serem adotadas. Troy et al. demonstraram que apenas 9% dos médicos se sentiam confortáveis em tratar atletas com a tríade,^(16)^ o que nos alerta sobre a necessidade de se divulgar a informação entre os profissionais envolvidos no tratamento dessas mulheres.

A falta de protocolos adequados é a maior dificuldade e a maior crítica aos estudos que visam avaliar a prevalência de alterações ginecológicas e da tríade da mulher atleta entre mulheres esportistas. As outras particularidades, como a incontinência urinária, a TPM e a influência do ciclo menstrual sobre o desempenho esportivo, entretanto, também estão suscetíveis às mesmas críticas e à falta de explicações definitivas, sendo, usualmente, pouco valorizadas. Esse fato deve, assim, confirmar a importância da investigação ativa e de intervenções precoces que possam orientar e garantir a saúde dessas meninas.

Este estudo não teve como objetivo, efetivamente, a comparação estatística entre modalidades, pois o número de participantes em cada grupo não se mostrou uniforme. As análises foram, entretanto, realizadas e mencionadas, quando pertinentes.

Este estudo reitera que as questões ginecológicas e as particularidades da mulher atleta devem ser ativamente investigadas e não subestimadas durante seu acompanhamento, por serem relevantes à sua saúde e, consequentemente, ao seu rendimento esportivo.

## CONCLUSÃO

O instrumento aqui proposto, denominado *Pre-Participation* *Gynecological Examination*, permitiu a identificação de questões usualmente não referidas durante a avaliação pré-participação convencional.

O cuidado específico da mulher atleta, por vezes subestimado, demonstrou-se de extrema importância e o instrumento proposto pode ser de grande valia para o rastreamento de alterações particulares dessa população. O *Pre-Participation Gynecological Examination* pode ser aplicado durante a realização da avaliação pré-participação feminina, de maneira rotineira, pelo médico responsável, identificando alterações e permitindo, assim, o encaminhamento para profissional especializado.

## Appendix

**Anexo 1:** *Pre-Participation Gynecological Examination* (PPGE)

Dados gerais
1) Data de nascimento (D/M/A): __________Peso_______ Altura______
Raça (_) Branca__(_) Não branca
2) Escolaridade
(_) Ensino Fundamental (1^a^ – 8^a^ série)__(_) Ensino Médio (colegial)
(_) Ensino Superior__(_) Completo__(_) Incompleto curso:_____________________
**Histórico esportivo**
3) Modalidade: ____________
4) Idade de início de treinamento competitivo: _______ anos
5) Horas de treino por dia atualmente: ______ horas
6) Frequência semanal de treinos atualmente:__________ dias/semana
**Antecedentes menstruais**
7) Idade da primeira menstruação: ______ anos
8) Treina menstruada? (_) Sim__(_) Não
9) Quantos dias dura sua menstruação? _____ dias
10) Depois de quanto tempo você menstrua novamente?
(_) não consigo saber porque varia muito
(_) não consigo saber mas menstruo uma vez por mês
(_) Depois de ____ dias
11) Já ficou 3 meses (ou mais) sem menstruar? (_) Sim__(_) Não
12) Se sim: foi em período de treinamento intenso? (_) Sim__(_) Não
estava com o peso abaixo do seu normal? (_) Sim__(_) Não
voltou ao normal quando diminuiu o ritmo de treino? (_) Sim__(_) Não
voltou ao normal quando ganhou peso? (_) Sim__(_) Não
não está associado ao treino ou alteração de peso (_)
**Influência do ciclo menstrual sobre o desempenho**
13) Seu técnico sabe quando você está menstruada? (_) Sim__(_) Não
14) Seu técnico aceita mudar seu treino se a menstruação estiver atrapalhando seu rendimento? (_) Sim__(_) Não__(_) Não falo nem peço isso pra ele(a)
15) Se pudesse escolher uma época para competir, você escolheria:
(_) O período menstrual não me influencia__(_) Menos durante a menstruação
(_) Logo antes da menstruação__(_) Durante a menstruação
(_) Logo após a menstruação__(_) Mais ou menos 15 dias após a menstruação
**TPM**
16) O que você sente antes de menstruar? Dê nota 1 (se incomoda pouco) ou 2 (se incomoda muito).
(_) Não sinto nada que realmente me atrapalhe __
(_) Dor ou inchaço nas mamas __
(_) Inchaço na barriga, sensação de peso ou desconforto __
(_) Dor de cabeça __
(_) Inchaço nas mãos ou nas pernas __
(_) Aumento de peso __
(_) Dor nas costas, nas juntas ou nos músculos __
(_) Irritação, nervosismo ou tristeza __
(_) Aumento de apetite __
(_) Alteração do sono __
(_) Falta de concentração, perda da motivação __
(_) Cólica menstrual __
**Antecedentes sexuais**
17) Você já teve relação sexual com homens? (_) Sim__(_) Não
18) Se sim, quantos anos você tinha na sua “primeira vez”? _______ anos
19) Quantos parceiros você já teve? _______
20) Tem parceiro fixo (tem relação só com essa pessoa)? (_) Sim__(_) Não
21) Se “sim”, há quanto tempo? __________
22) Já teve relação com mulheres? (_) Sim__(_) Não
23) Você usa algum método anticoncepcional? (_) Sim__(_) Não
24) Se “sim”, assinale qual:
(_) Pílula:__________(_) DIU progesterona__(_) Camisinha masculina
(_) Injeção mensal__(_) DIU cobre__(_) Camisinha feminina
(_) Injeção trimestral__(_) Anel vaginal__(_) Outro_______________
25) Foi prescrito por um ginecologista? (_) Sim__(_) Não
26) Começou a usar para: (_) Evitar gravidez__(_) Regular a menstruação__(_) Pelos dois motivos
27) Você sabe o que é DST? (_) Sim__(_) Não
28) Já teve que tratar alguma doença sexualmente transmissível? (_) Sim__(_) Não
29) Se “sim”:
Qual foi a doença? ___________________________________
Qual foi o tratamento? ________________________________
Teve cura? (_) Sim__(_) Não
**Antecedentes obstétricos**
30) Já engravidou?: Sim (_)__Não (_)__Quantas vezes? ___________
Número de partos (coloque o número): (_) Normal__(_) Cesárea__(_) Fórceps
Número de abortos (coloque o número): (_) Espontâneo__(_) Provocado
**Tríade da mulher atleta**
31) Já ouviu falar na “Tríade da Mulher Atleta”? Sim (_)__Não (_) __________
32) Já teve fratura por estresse? (fratura não associada a trauma, acidente ou torção aguda.) (_) Sim__(_) Não
33) Se “sim”, naquele período:
Estava menstruando normalmente? (_) Sim__(_) Não__(_) Não lembro
Tinha perdido peso? (_) Sim _______ kg__(_) Não__(_) Não lembro
Estava em período de treinamento intenso? (_) Sim__(_) Não__(_) Não lembro
Em que região do corpo ela ocorreu? __________________________
**Antecedentes pessoais**
34) Trata alguma doença? (_) Sim__(_) Não.__Qual?_________________________
35) Toma algum remédio regularmente? (_) Sim__(_) Não.__Qual?_______________
36) Fez alguma cirurgia? (_) Sim__(_) Não.__Qual?_____________Quando? ________
37) Tem alergia a algum alimento ou medicação? (_) Sim__(_) Não
38) Se “sim”, a que? _______________________________________
39) Você tem acompanhamento ginecológico de rotina (pelo menos anual)? (_) Sim__(_) Não
